# Simultaneous Occurrence of Nasal Carcinoma Induced by Enzootic Nasal Tumour Virus‐2 (ENTV‐2) and Border Disease Virus in Three Goats

**DOI:** 10.1002/vms3.70325

**Published:** 2025-04-17

**Authors:** Canan Akdeniz Incili, Yesari Eroksuz, Mehmet Ozkan Timurkan, Burak Karabulut, Zeynep Yerlikaya, Hatice Eroksuz

**Affiliations:** ^1^ Department of Pathology Faculty of Veterinary Medicine Firat University Elazig Turkey; ^2^ Department of Virology Faculty of Veterinary Medicine Ataturk University Erzurum Turkey; ^3^ Department of Microbiology Faculty of Veterinary Medicine Firat University Elazig Turkey

**Keywords:** border disease virus, enzootic nasal tumour virus‐2, histopathology, immunohistochemistry, PCR

## Abstract

**Background:**

Enzootic nasal adenocarcinoma (ENA) is a prevalent neoplastic disease affecting small ruminant populations globally, caused by the enzootic nasal tumour virus (ENTV). Border disease (BD), also affecting small ruminants, causes persistent infection and increases secondary infection risks.

**Methods:**

Post‐necropsy tissue samples were routinely processed and stained with haematoxylin and eosin. Histopathological, immunohistochemical and PCR techniques were employed to diagnose ENTV‐2 in three goats. Viral RNA extraction and reverse transcription were performed using the WizPrep Viral DNA/RNA micro kit. PCR analysis targeted ENTV‐1, ENTV‐2 and BD virus (BDV).

**Results:**

ENTV‐2‐induced nasal carcinoma and BDV were identified in three goats from an Eastern Turkey breeding facility. Post‐mortem examinations revealed neoplastic growths originating from the ethmoid region and extending throughout the nasal cavity. PCR analysis revealed the presence of both ENTV‐2 and BDV genomes in all three cases. Histopathological examination showed invasive acinar and papillary proliferations with multifocal necrosis and lymphohistiocytic infiltrations. Immunohistochemical analysis demonstrated positive reactivity for ENTV in neoplastic cells using a monoclonal antibody specific to the JSRV capsid protein, which also cross‐reacts with the envelope protein of ENTV‐1.

**Conclusion:**

The chronic, immune‐tolerant nature of BD infection may have immunosuppressive effects that potentially enhance the impact of ENTV. The simultaneous presence of these two pathogens could synergistically increase the risk of morbidity and mortality in caprine populations.

## Graphical abstract

ENTV‐2‐induced nasal carcinoma and BD virus were identified in three goats within a breeding facility in Turkey. Post‐mortem examinations showed neoplastic growths originating predominantly from the ethmoid region and extending throughout the nasal cavity. Histopathological examination revealed acinar and papillary proliferations displaying invasive characteristics together with multifocal necrosis and lymphohistiocytic infiltrations. PCR analysis identified concurrent genomes of ENTV‐2 and BDV in all examined cases. Their concurrent presence poses a significant risk and elevating the mortality among goats.

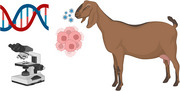

## 1 Introduction

Enzootic nasal adenocarcinoma (ENA) is a globally contagious neoplastic disease affecting small ruminant populations, with the exception of New Zealand and Australia. The etiological agent, enzootic nasal tumour virus (ENTV), is classified under the *Betaretrovirus* genus within the Retroviridae family. ENTV primarily induces localised tumours in the nasal passages of sheep and goats. Two subtypes are recognised: ENTV‐1, predominantly affecting sheep, and ENTV‐2, more common in goats (Walsh et al. 2013). ENVT has a strong phylogenetic similarity (amino acid and nucleotide similarity) to the Jaagsiekte sheep retrovirus (JSRV), also within the *Betaretrovirus* genus of the Retroviridae family (De las Heras et al. 2003). Therefore, the distinction between these two infections is made genetically through phylogenetic analyses.

Epidemiological studies have demonstrated that ENTV prevalence ranges from 0.1% to 15% in affected populations, primarily impacting young adults aged 3–5 years. Notably, the disease exhibits no apparent genetic, gender, or breed predisposition (De las Heras et al. 1998, 2019; DeMartini and York 1997). At the molecular level, ENTV activates the PI‐3K/Akt signaling pathway, which is crucial for cell proliferation and survival in cancer development. The tyrosine residue at position 590 in the ENTV envelope protein plays a critical role in this process (Maeda and Fan 2008). Although the adenocarcinoma associated with ENA rarely metastasises due to its low‐grade characteristics, its invasive growth pattern can cause local damage to anatomical structures such as the nasal mucosa, nasal septum and cribriform plate, and also infiltrate surrounding regions such as the nasopharynx and sinuses (De las Heras et al. 1995).

Border disease (BD), another globally prevalent condition, is characterised by immunosuppression and reproductive impairment, predominantly affecting sheep and occasionally goats. The clinical manifestations of BD encompass four distinct disease syndromes: early embryonic death, abortion and stillbirth, birth of lambs with malformations and birth of small, weak lambs. Surviving lambs frequently succumb shortly after birth, although some gradually recover (Mishra et al. 2016; Righi et al. 2021). Survivors and apparently healthy lambs, may become persistently infected with the virus, continuously excreting it throughout their lifespan. These persistently infected sheep play a crucial role in the epidemiology of BD and potentially serve as a source of infection for susceptible populations (Nettleton 1987; Righi et al. 2021). The causative agent of BD belongs to the genus *Pestivirus* within the family Flaviviridae. This genus comprises four recognised species: Bovine viral diarrhoea virus 1 (BVDV‐1), bovine viral diarrhoea virus 2 (BVDV‐2), border disease virus (BDV) and classical swine fever virus (CSFV) (Mishra et al. 2016; Righi et al. 2021). Persistent infections, particularly when combined with immune suppression, can facilitate mixed or coinfections. BD has been extensively studied, especially in relation to its serological and virological characteristics. The primary site of BD pathology in pregnant adult sheep is the placenta, where it causes necrotic carunculitis (Nettleton 1987). Previous studies in Turkey have provided significant histological and electron microscopic findings on ENA in goats and sheep (Özmen et al. 2010; Özmen and Serpin 2016). However, no molecular or immunohistochemical studies have been conducted in the country. This study examines the pathological, immunohistochemical and molecular characteristics of concurrent ENA and BD in goats.

## 2 Materials and Methods

### 2.1 Histopathological and Immunohistochemical Methods

Following the necropsy of each case, tissue samples from the tumour tissue, lungs, cardiac muscle, liver, brain, kidneys, large and small intestines were collected, fixed in 10% formalin, and paraffin‐embedded and sectioned at 4–5 µm thickness. Subsequently, they were stained with haematoxylin and eosin (H&E). Immunostaining involved the avidin‐biotin complex method (Hsu et al. 1981). Immunohistochemical detection of the ENTV envelope protein was performed using a monoclonal antibody against the JSRV envelope protein. This antibody shows cross‐reactivity with the envelope protein of ENTV‐1, allowing for effective identification of associated viral antigens. The antibody was diluted 1:200 and incubated with anti‐polyvalent antibodies for 60 min at 37°C. Immunodetection utilised a peroxidase‐labelled streptavidin and a labelled streptavidin‐biotin kit, with 3‐Amino‐9‐ethylcarbazole (AEC) as the chromogenic substrate. Slides were counterstained with Mayer's haematoxylin for visualization. Archived lung tissue sections previously confirmed positive for JSRV via polymerase chain reaction (PCR) and pathological analysis (Can‐Sahna et al. 2015; Coskun et al. 2024) served as positive controls. Normal goat nasal mucosa with standard histological features was used as the negative control.

### 2.2 Virological Examination

#### 2.2.1 Sample Preparation

The extraction procedure was initially applied to process neoplastic and other (brain, liver and spleen) organ samples. Tissue specimens underwent mechanical trimming using a scalpel and scissors. Following that, the tissue was further reduced and homogenised for 5 min at 4000 rpm using a tissue homogeniser (Tissue Lyser LT, Qiagen, Germany) and a lysis buffer. Subsequently, the homogenised tissue was centrifuged at 10,000 rpm for 5 min. After centrifugation, 200 µL of the supernatant was collected to proceed with the extraction procedure.

#### 2.2.2 Extraction of Viral Nucleic Acid

Viral RNA was isolated using a versatile nucleic acid extraction kit capable of handling both types of nucleic acids. Specifically, the WizPrep Viral DNA/RNA micro kit (WizBio in South Korea) was utilised for this process. Following that, the extracted material was stored at −20°C pending the analytical procedures.

#### 2.2.3 Reverse Transcription of Viral RNA (RT)

In the case of RNA viruses, specifically ENTV (from the Retroviridae family, *Betaretrovirus* genus) and BDV (from the Flaviviridae family, *Pestivirus* genus), viral RNA was acquired through the extraction of viral nucleic acid. Following extraction, the obtained RNA was utilised as a template for complementary DNA (cDNA) synthesis. For this purpose, a commercially available first strand cDNA synthesis kit, which includes the reverse transcriptase enzyme (Thermo Fisher Scientific, USA), was utilised. This kit also includes a DNAase enzyme. For each sample, a mixture for cDNA synthesis was prepared, comprising 3 µL sterile distilled water, 0.5 µL random hexamer primer (Random Hexamer, Thermo Fisher Scientific, USA) and 3 µL of the RNA‐containing mixture. Subsequently, the tubes were positioned in a thermal cycler (Applied Biosystems, Verity, USA). In the initial step, the tubes containing the extracted RNA were subjected to a temperature of 70°C for 5 min, followed by rapid cooling on ice. Subsequently, for the second step, a second mixture was prepared, composed of 2.0 µL of ×5 reaction buffer, 1.0 µL of 10 mM dNTP mix and 0.5 µL of reverse transcriptase. This second mixture was gently added to the tubes containing the first mixture, and the reaction proceeded at 25°C for 10 min, followed by 37°C for 60 min and concluded with a final incubation at 70°C for 5 min. At the end of this procedure, the cDNA was effectively synthesised and obtained from the RNA template. The resulting cDNA was then utilised as the template for the subsequent PCR process.

### 2.3 PCR Analysis of Viral Pathogens

#### 2.3.1 ENTV

After the reverse transcription, each sample underwent distinct PCR analysis for both ENTV‐1 and ENTV‐2 to amplify the nucleic acids. ENTV‐1 long repeat region gene (LTR) and ENTV‐2 envelope gene primers were selected. A 40‐cycle PCR process was applied using ENTV‐1‐F (forward) (5′‐ AAGCAAGTTAAGTAACTTGAGATC‐3′) and ENTV‐1‐R (reverse) (5′‐ GCTTAGCCGTCCTAAAAGAG‐3′) primers, which are specific to the ENTV‐1 LTR gene region. And ENTV‐2‐F (forward) (5′‐ AGCTGCTCATACTGTGGATC ‐3′) and ENTV‐2‐R (reverse) (5′‐ GATCTTATCTGCTTATTTTCAG ‐3′) primers, which are specific to the ENTV‐2 envelope gene region (Table 1). The PCR programs applied in the thermal cycler device were as follows: denaturation at 94°C for 5 min, 94°C for 1 min, 55°C for ENTV‐1 and 53°C for ENTV‐2 for 1 min and 72°C for 1 min. After a final extension step of 5 min at 72°C, no positivity was obtained for ENTV‐1 (since ENTV‐1 positivity was not detected, the PCR gel was not added to the official study), but an amplification product of 822 bp was obtained for ENTV‐2 (Figure 3A). Retroviruses have exogenous and endogenous characters. However, when there is a visible necropsy detection and symptoms in the study, the positivity determination indicates an exogenous character. In the PCR reactions, we used samples provided by our department as positive controls (Table 1).

**TABLE 1:** Virus, gene, PCR primers and PCR products.

Virus/gene	Primers	PCR product (bp)/ annealing extension °C
**Pan Pestiviruses (5’UTR gene region)**	324‐ATGCCCWTAGTAGGACTAGCA 326‐TCAACTCCATGTGCCATGTAC	288 bp (56°C)
**Border disease specific PCR (5’UTR)**	PBD1‐ TCGTGGTGAGATCCCTGAG PBD2‐ GCAGAGATTTTTTATACTAGCCTATRC	225 bp (56°C)
**ENTV‐1 (LTR gene)**	ENTV‐1F AAGCAAGTTAAGTAACTTGAGATC ENTV‐1R GCTTAGCCGTCCTAAAAGAG	938 bp (ENTV‐1, 55°C)
**ENTV‐2 (envelope gene)**	ENTV‐2F‐AGCTGCTCATACTGTGGATC ENTV‐2R GATCTTATCTGCTTATTTTCAG	822 bp (ENTV‐2, 55°C)

#### 2.3.2 BD Virus

After the reverse transcription, all samples were analysed using PCR to detect the presence of the pestiviruses. The 5’UTR (5’ untranslated region) primer was selected for the pestiviruses (Table 1). A PCR process was applied using panpesti primer 324 (forward) (5′ ATGCCCWTAGTAGGACTAGCA 3’) and panpesti primer 326 (reverse) (5’ TCAACTCCATGTGCCATGTAC 3’) (Timurkan and Aydın 2019) (Figure 3B). In the RT‐PCR, we employed the primers and optimisation conditions for BVDV as described earlier (Timurkan and Aydın 2019). After *Pestivirus* positivity was detected, PCR analysis was performed again with BD specific primers (primers: PBD1 and PBD2), even though a *Pestivirus* detected in goats was reminiscent of BDV (Figure 3C) (Oguzoglu et al. 2009). The thermal cycling conditions and primers utilised in the reaction were chosen in accordance with references cited in the literature. The resulting amplicons from the PCR were subsequently assessed through gel electrophoresis.

#### 2.3.3 Sequence Analysis of ENTV

After PCR, sequence analysis was performed to reveal the genomic differences of ENTV and JSRV in the *Betaretrovirus* genus. Phylogenetic analysis was performed using MEGA 11 software with the raw data obtained after the sequencing reaction and reference strains for ENTV, JSRV and other retroviral infections obtained from the gene bank.

## 3 Results

### 3.1 Herd History and Clinical Presentation

This study examined three goats from a breeding unit that comprised 150 adult goats and 20 kids. The animals were transported to the department of pathology on separate occasions. In the herd, 15 out of 150 animals (10%) showed clinical signs. These signs included anorexia, coughing, nasal discharge and emaciation, primarily observed in animals aged 2 years or older. Importantly, one 6‐month‐old animal also presented with similar signs, representing an exception to the age‐related pattern.

### 3.2 Gross Findings

**FIGURE 1:**
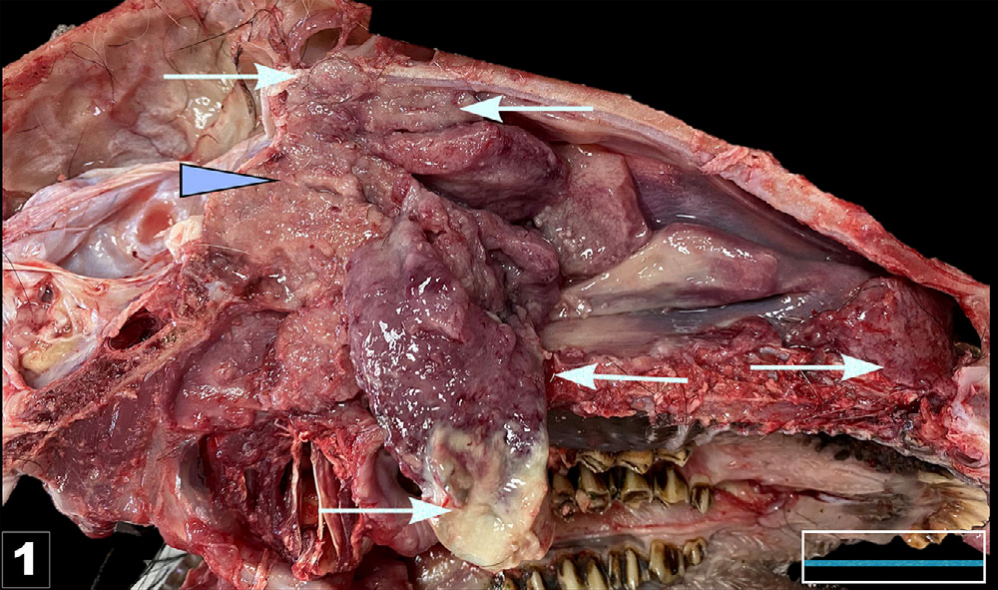
The tumour originating in the ethmoidal bone (the arrow head) extended into and nearly completely occupied the nasal cavity (arrows) in the sagittal section of the cranium, Bar: 3 cm.

Necropsy examinations of all three goats revealed neoplastic formations within the nasal passages, predominantly concentrated in the ethmoidal region. These tumours exhibited invasive progression in all directions (Figure 1). The neoplastic masses presented as friable, greyish–white or fleshy growths, causing significant damage to the nasal turbinates. In addition, thin and often incomplete fissures, measuring 2–3 mm in diameter, were observed near the median line of the nasal bone. In one goat, a dead *Oestrus ovis* larva was present within the mass. The examination of other organs revealed no signs of metastasis.

### 3.3 Histopathological Findings

**FIGURE 2:**
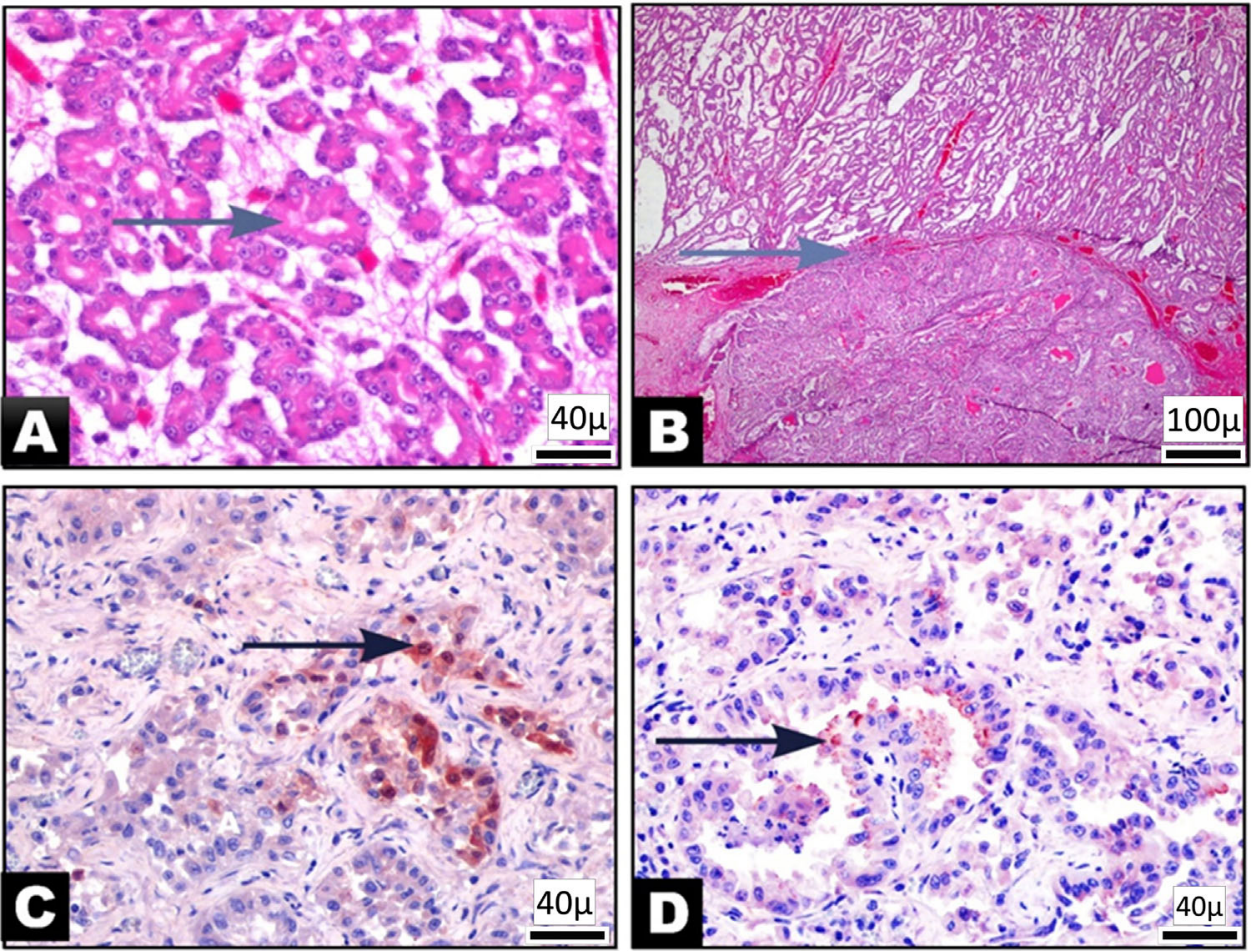
A–D: Histologic and immunohistochemical characteristics of the tumour. (A) Neoplastic cells forming the acinar structures (arrow), X40, Bar: 20 µm, H&E. (B) Fine fibrous septa between the acianar and tubular proliferations, X4, Bar: 200 µm, H&E. (C, D). Neoplastic cells showed focally extensive intense to marked red staining (arrows) to rabbit monoclonal antibody raised against the capsid protein of, JSRV; counterstain Mayer haematoxylin, ABC immunohistochemistry, X40, Bar: 20 µm.

In all three cases, microscopic examinations consistently demonstrated uniform histopathological characteristics of nasal adenocarcinoma. The findings were marked by the proliferation of mucous and serous glandular cells in an acinar pattern (Figure 2A). In addition, these neoplastic cells also displayed papillary structures, which were accompanied by delicate fibrous septa interposed between them (Figure 2B). They had a columnar or cuboidal shape with varying degrees of distinctive eosinophilic cytoplasm. The nuclei of the tumour cells exhibited either oval vesicular or round hyperchromatic characteristics. Those with vesicular nuclei displayed a central nucleolus and numerous strippled chromatin granules. Notably, tumour cells with vesicular nuclei showed a higher degree of differentiation compared to those with round hyperchromatic nuclei. Despite the cells exhibiting limited nuclear pleomorphism and few mitotic figures, a notable infiltration of adjacent tissues was evident. The stroma was edematous and contained focal necrotic foci.

As associated lesions; turbinated bone in the coancha showed a heterogeneous pattern of necrosis, interspersed with areas exhibiting active remodelling and bone resorption. Multifocal lymphohistiocytic cell infiltrations were present within the tumour tissue and in the stromal regions of adjacent intact mucosa. The blood vessels were markedly congested.

The extranasal lesions include lymphoid depletion in the spleen and lymph nodes in all three animals. Moderate interstitial pneumonia (1/3), and portal lymphohistiocytic infiltration (2/3).

### 3.4 Immunonohistochemical Findings

Positive immunostaining for ENTV was detected in the cytoplasm of neoplastic cells and infiltrated macrophages, showing a multifocal distribution (Figure 2C,D).

### 3.5 Virological Results

**FIGURE 3:**
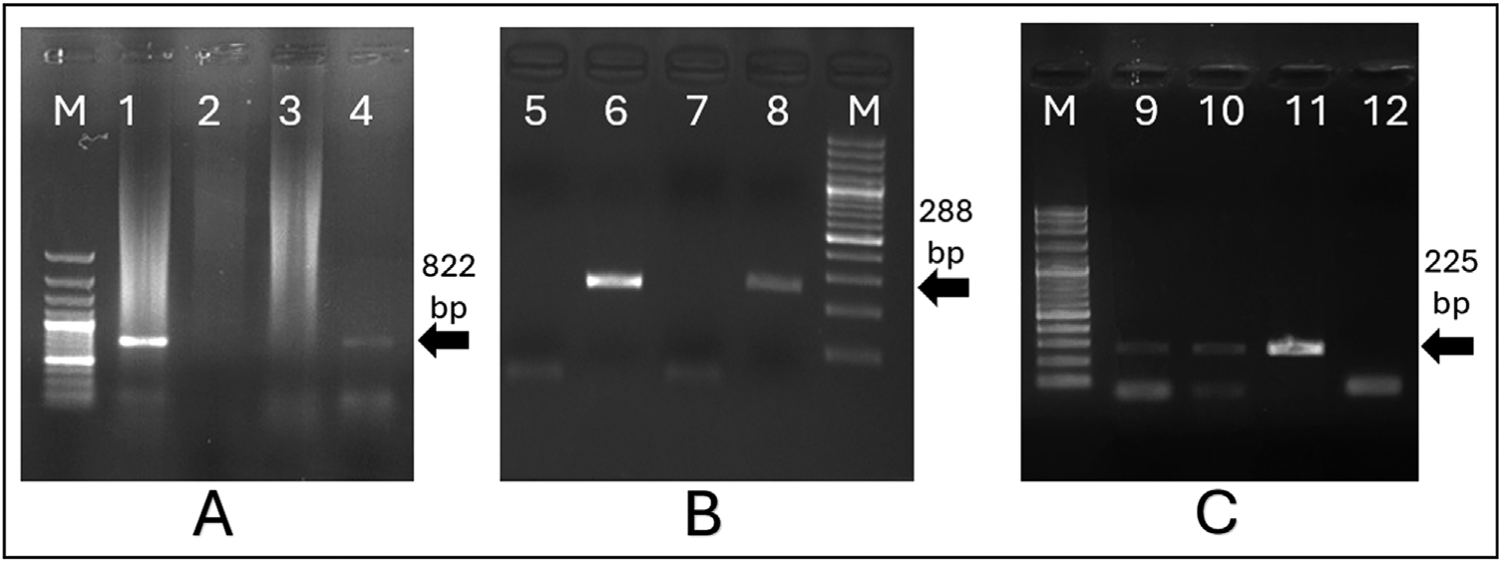
PCR agarose gel image. (A) ENTV‐2 positivity, M: DNA ladder (100 bp), 1: Positive control (822 bp), 2: Negative control, 3: Negative field sample, 4: Positive field sample. (B) Pestivirus positivity, 5: Negative control, 6: Positive control (288 bp), 7: Negative field sample, 8: Positive field sample. (C) Border disease positivity, 9–10: Positive field sample, 11: Positive control (225 bp), 12: Negative control.

Firstly, the presence of ENTV was investigated in the samples included in the study, and while no positivity was detected for ENTV‐1, positivity for ENTV‐2 was detected (Figure 3A). Later, a scan was performed for BD. For this purpose, *Pestivirus* genus scanning was performed. Because the *Pestivirus* genus detection is used as a precursor for BD,

*Pestivirus* positivity was also detected in the screening (Figure 3B). Then, PCR was performed with BD‐specific primers to determine that *Pestivirus* positivity was caused by BD and positivity was detected (Figure 3C). In the PCR agarose images detailed in Figure 3, both positive sample (positive sample distributed randomly in the study) and positive and negative controls can be seen.

**FIGURE 4:**
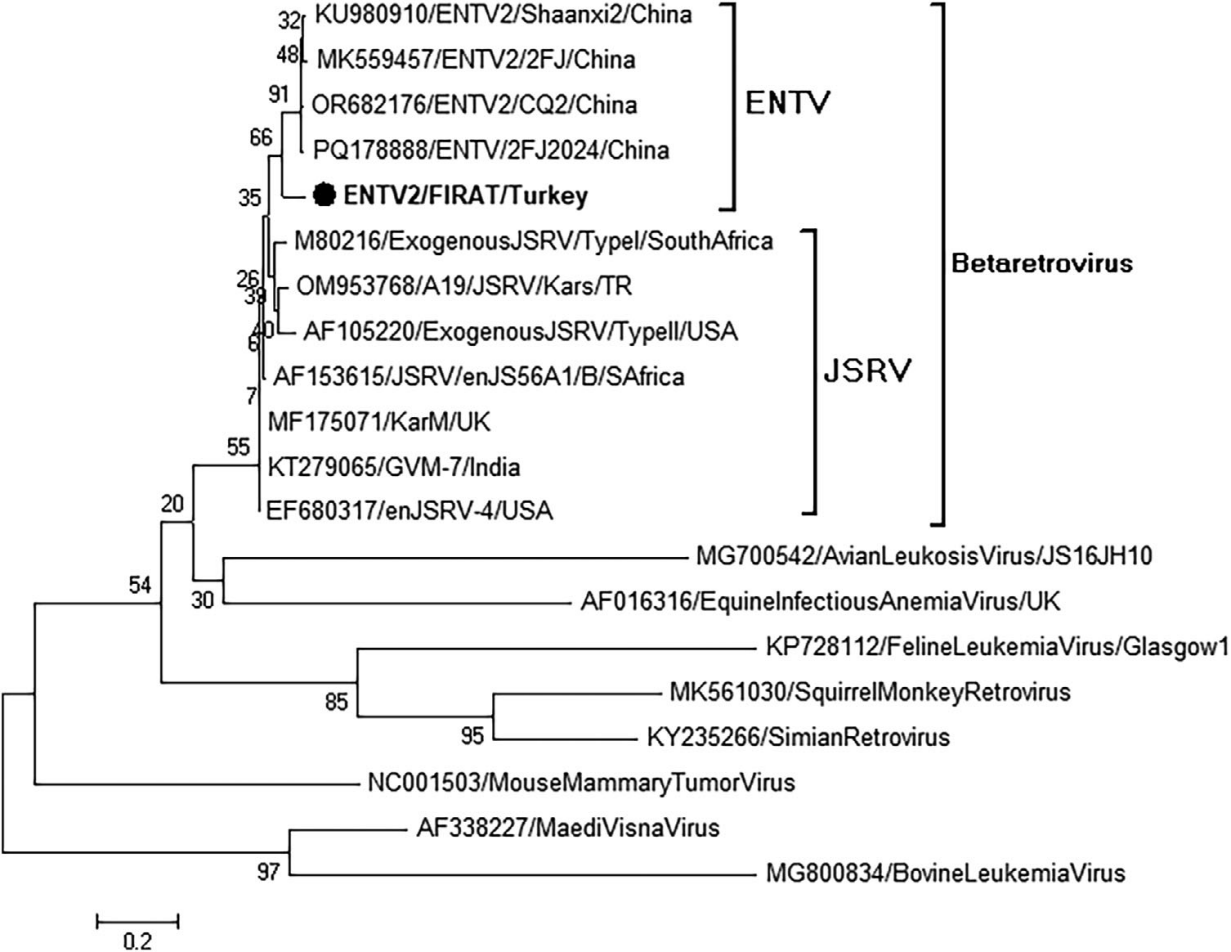
Phylogenetic tree constructed using reference strains and our isolate. Black circle is the ENTV Turkish strain of this study.

The sequence of the strain obtained in the study was brought together with reference strains for ENTV, JSRV and other retroviral infections obtained from the gene bank for phylogenetic analysis. Phylogenetic analysis was performed using MEGA 11 software. As a result of the analysis, it was determined that the strain obtained was phylogenetically in the same cluster with ENTV‐2 strains and clustered separately from JSRV strains at the genomic level (Figure 4).

## 4 Discussion

ENA is a neoplastic condition affecting small ruminants globally (Li et al. 2022). While serological tests are available, their reliability may be compromised due to limited antibody production against the viral capsid (Walsh et al. 2013). Most ENA reports have primarily utilised histopathological findings and electron microscopic detection of virions (Özmen et al. 2010; Özmen and Serpin 2016; DeMartini and York 1997; Vitellozzi et al. 1993; McKinnon et al. 1982), with fewer studies incorporating molecular techniques (Delaude et al. 2020; Jahns and Cousens 2020; Ye et al. 2019). However, the combination of PCR enables etiological diagnosis (Jahns and Cousens 2020). Our immunohistochemical findings corroborate previous studies (Jahns and Cousens 2020; Stowe et al. 2012; Walsh et al. 2013), demonstrating cross‐reactivity between antibodies targeting the JSRV capsid protein and ENTV. Notably, a prior case report documented the induction of spontaneous nasal adenocarcinoma by JSRV (Jahns and Cousens 2020). This observation underscores the potential interplay between these related viruses in oncogenesis.

The concurrent infection with BDV, a *Pestivirus*, adds complexity to the case. BDV invasion and replication in lymphocytes and macrophages can lead to both quantitative and functional declines in these cells, compromising the immune system's ability to respond effectively to infections (Oguzoglu 2018; Righi et al. 2021). Furthermore, viral interference with cytokine production exacerbates immune dysregulation (Oguzoglu 2018). The importance of preventing persistent infections, including those caused by pestiviruses, and addressing coinfections in small ruminant populations cannot be overstated (Righi et al. 2021; Oguzoglu 2018). Our study identified a herd with infections from both retrovirus and *Pestivirus*. Previous reports of BDV coinfections with various pathogens, including *Chlamydophila abortus* (Şevik 2023), peste des petits ruminants virus (Kul et al. 2008) and several bacterial agents (Şevik et al. 2017; Righi et al. 2021), strongly support BDV's immunosuppressive capabilities. With BD seroprevalence ranging from 30% to 98% (Righi et al. 2021), the risk is substantial (Righi et al. 2021).

To our knowledge, this is the first report of simultaneous ENA and BDV infections, suggesting a synergistic effect on disease severity and mortality in goats. As a result, sheep and goats are a herd‐based animal population. Therefore, protecting herd health will be the main goal for the animal farm. In our study, the ENTV infection detected alongside the BD infection that will cause a herd problem is, as far as we know, the first detection. Therefore, the presence and prevalence of this infection should now be examined in herd screenings. The detection of these infections (such as retrovirus and *Flavivirus* infections) together, especially those with the potential to suppress the immune system, is also important data. Such studies are needed in the future with a broader perspective.

## Author Contributions

**Canan Akdeniz Incili**: investigation, methodology, visualisation, writing – original draft. **Yesari Eroksuz**: data curation, formal analysis, investigation, methodology, supervision, writing – original draft. **Mehmet Timurkan**: investigation, methodology, visualisation. **Burak Karabulut**: investigation, methodology, visualisation. **Zeynep Yerlikaya**: data curation, formal analysis, investigation, methodology. **Hatice Eroksuz**: supervision, visualisation, writing – original draft.

## Ethics Statement

The authors have nothing to report.

## Consent

We give our consent for the publication of identifiable details, which can include photograph(s) and/or videos and/or case history and/or details within the text (“Material”) to be published in the above journal and article.

## Conflicts of Interest

The authors declare no conflicts of interest.

### Peer Review

The peer review history for this article is available at https://www.webofscience.com/api/gateway/wos/peer‐review/10.1002/vms3.70325

## Data Availability

The authors confirm that the data supporting the findings of this study are available within the article [and/or] its supplementary materials.
